# Complete Genome Sequences of Two Vibrio natriegens Bacteriophages

**DOI:** 10.1128/MRA.01133-20

**Published:** 2020-11-05

**Authors:** Meghan T. Harris, Tereasa Ching Ho, Harry Fruchtman, Mira E. Garin, Victor Kubatin, Tiger Lu, Lingyan Xue, Michael T. Marr

**Affiliations:** a Department of Biology, Brandeis University, Waltham, Massachusetts, USA; b Rosenstiel Basic Medical Sciences Research Center, Waltham, Massachusetts, USA; DOE Joint Genome Institute

## Abstract

Vibrio natriegens, a fast-growing Gram-negative bacterium, is gaining interest as a platform for rapid biotechnology applications and metabolic engineering. Only a few bacteriophages that infect this bacterium have been identified. Here, we describe the isolation and characterization of two *V. natriegens* bacteriophages isolated from Hatches Creek, Wellfleet, Massachusetts.

## ANNOUNCEMENT

Vibrio natriegens is a Gram-negative moderately halophilic marine bacterium first isolated in the 1950s (1). With a doubling time of less than 10 minutes under optimal conditions, it is the fastest-growing bacterium (2). It has garnered interest as a biotechnology platform for protein production, molecular biology, and metabolic engineering (3–6). Identifying bacteriophage that infect *V. natriegens* has the potential to aid in these efforts, as molecular genetic tools are often derived from bacteriophage (7).

Environmental samples from Cape Cod, Massachusetts, were tested for bacteriophage by spotting them onto Luria-Bertani *Vibrio natriegens* broth (LB-Vn) top agar (1% tryptone, 0.5% yeast extract, 3% NaCl, 4 mM KCl, 20 mM MgSO_4_, 4 mM CaCl_2_, and 0.5% agar) containing *V. natriegens* (ATCC 14048). *V. natriegens* was prepared by a 1:100 dilution of an overnight culture in LB-Vn liquid medium, followed by aerobic growth at 37°C for 4 to 6 hours. Zones of clearing were observed for one sample of filtered water from a mixture of sediment and brackish water taken from Hatches Creek in Wellfleet, Massachusetts. The clear spot was harvested by scraping and then suspending it in phage buffer (50 mM Tris [pH 7.5], 100 mM NaCl, 8 mM MgSO_4_, 5 mM CaCl_2_, and 0.01% gelatin). Individual plaques were obtained by serial dilution of this sample in phage buffer and two rounds of plaque purification on LB-Vn top agar plates containing *V. natriegens*.

Genome sequences were obtained by a combination of methods. Two well-separated plaques were subjected to direct sequencing using a Nextera kit (8). Phage genomic DNA was also purified from high-titer stocks prepared from webbed plates using the Promega Wizard DNA cleanup system followed by Nextera tagmentation. Libraries prepared directly from plaques were sequenced on a NextSeq 500 instrument with a 75-cycle output. Libraries created from purified DNA were sequenced on a MiSeq instrument using 150-bp reads. Libraries were initially sequenced and analyzed individually. All three libraries yielded the same two phage genomes. To increase the confidence and coverage of the genomes, the library sequences were combined and reassembled. The combined library contains 140,432,773 total reads. Reads were processed online using Galaxy and the Center for Phage Technology (CPT) Galaxy. Reads were quality checked and trimmed using FastQC (Galaxy version 0.72+galaxy1) and Trim Galore! (Galaxy version 0.6.3). Trimmed reads were aligned to the *V. natriegens* genome (9) using Bowtie 2 (Galaxy version 2.3.4.3+galaxy0) (10). Unaligned reads were used to assemble the bacteriophage genome sequences using Unicycler (Galaxy version 0.4.8.0) (11). The Unicycler output contained two separate scaffolds. Both scaffolds were considered to represent complete bacteriophage genomes based on the following two lines of evidence: (i) the Unicycler output flagged both genomes as “circular,” indicating overlapping reads across the entire scaffold, and (ii) manual permutation and read mapping with Bowtie 2 showed consistent coverage across the original scaffold origin. The genomes were annotated using Prokka (Galaxy version 1.14.5) (12).

The genome of bacteriophage VH1_2019 is 246,059 bp with a GC content of 42.6%. The sequence was assembled with an average base coverage of 31,443×. It is a member of the T4 *Myoviridae* family. The genome contains 381 open reading frames (ORFs) and 27 tRNA genes. BLASTn searches using NCBI Virus indicate that VH1_2019 is closely related to the broad-host-range vibriophage KVP40 (13). The two viruses are 98% identical over 97% of the length of the VH1_2019 sequence.

The genome of bacteriophage VH2_2019 is 81,721 bp with a GC content of 48%. It was sequenced with an average base coverage of 2,318×. It is a member of the *Siphoviridae* family. The genome contains 108 ORFs. NCBI Virus BLASTn analysis indicates that VH2_2019 is most closely related to vibriophage vB_VpS_PG28. The two viruses are 88% identical over 94% of the length of the VH2_2019 sequence.

### Data availability.

The annotated genome sequences for VH1_2019 and VH2_2019 were deposited under GenBank accession numbers MN794232 and MN794238, respectively. The raw sequencing reads were deposited in the Sequence Read Archive (SRA) under BioProject number PRJNA663890.
